# Scenario Simulation-Based Assessment of Trip Difficulty for Urban Residents under Rainstorm Waterlogging

**DOI:** 10.3390/ijerph9062057

**Published:** 2012-05-31

**Authors:** Peng Chen, Jiquan Zhang, Xinyu Jiang, Xingpeng Liu, Yulong Bao, Yingyue Sun

**Affiliations:** 1 College of Urban and Environmental Sciences, Natural Disaster Research Institute, Northeast Normal University, Changchun, Jilin 130024, China; Email: pp11290@163.com (P.C.); liuxp912@nenu.edu.cn (X.L.); baoyl751@nenu.edu.cn (Y.B.); 2 School of Travel and Geographical Sciences, Jilin Normal University, Siping, Jilin 136000, China; Email: ppsyy1129@163.com; 3 Disaster Prevention Research Institute, Kyoto University, Uji 611-0011, Japan; Email: jiangxy354@nenu.edu.cn

**Keywords:** scenario simulation, urban rainstorm waterlogging, GIS, trip difficulty, Ha-Erbin City

## Abstract

In this study, an experiment was performed to assess the trip difficulty for urban residents of different age groups walking in various depths of water, and the data were corroborated with the real urban rainstorm waterlogging scenarios in downtown (Daoli district) Ha-Erbin (China). Mathematical models of urban rainstorm waterlogging were constructed using scenario simulation methods, aided by the GIS spatial analysis technology and hydrodynamic analysis of the waterway systems in the study area. Then these models were used to evaluate the impact of waterlogging on the safety of residents walking in the affected area. Results are summarized as: (1) for an urban rainstorm waterlogging scenario reoccurring once every 10 years, three grid regions would have waterlogging above 0.5 m moving at a velocity of 1.5 m/s. Under this scenario, waterlogging would accumulate on traffic roads only in small areas, affecting the safety and mobility of residents walking in the neighborhood; (2) for an urban rainstorm waterlogging scenario reoccurring once every 20 years, 13 grids experienced the same waterlogging situation affecting a larger area of the city; (3) for an urban rainstorm waterlogging scenario reoccurring once every 50 years, 86 grid regions were affected (waterlogging above 0.5 m moving at 1.5 m/s), and those areas would become impassable for residents.

## 1. Introduction

Urban areas face potentially significant threats from both natural and human-made catastrophes. Cities growing rapidly at the current speed become very vulnerable to environmental problems, particularly the rainstorm waterlogging problem. As soon as rainwater starts to accumulate on roadways in urban areas, it will become a hazard for the residents because walking across 20 cm deep water is very difficult. Bikers and motorcycles would have problem passing through 30 cm deep water on roads. The most problematic spots are those under bridges as waterlogging can block traffic flow [1].

In the majority of cities, rainstorm waterlogging normally occurs within a very short period of time after heavy rainfalls, but rainstorms are not the only cause of waterlogging problems. As a result of the urbanization process, the topology, terrains, and water convergence conditions in urban areas are altered, leading to the decimation of vegetation coverage and river and pond areas. Large proportions of land have been changed into impervious areas, resulting in reduced ground water holding capacity, shortened water stagnation periods and low permeability, and fast surface runoff. On the other hand, the underground water discharge pipe network facilities are typically not upgraded concurrently with city development, so there is a lack of sufficient capacity for discharging such a large volume of water. These two factors together are responsible for causing urban rainstorm waterlogging disasters. 

The potential damage from waterlogging in urban area increases as the resident population continues to grow, and buildings are more and more compacted with highly centered economic activities. When a waterlogging disaster happens, the normal routines of daily life and the network of society activities are disrupted. When the commuting and transportation systems are interrupted as roads become impassable to residents and motor vehicles, and life support facilities residents will be inaccessible to residents, resulting in big threats to human life and property. Therefore, to accurately assessing the level of residents’ trip difficulty under rainstorm waterlogging conditions is very important for developing evacuation and emergency management plans during a disaster. 

When assessing the trip difficulty of urban residents, the best way is to use data recorded in previous urban rainstorm waterlogging events to construct mathematical models which are used to simulate the road conditions under various waterlogging scenarios. In developed countries where urbanization started very early, comprehensive strategies to manage urban waterlogging disasters have been developed and some of their experiences are very valuable [2,3].

From the late 19th to the mid-20th centuries, several very important and hydraulics-mathematical models (also including some hydraulic models) have been proposed consecutively, which are the theoretical basis for urban hydrology studies. The mathematical models include the de Saint-Venant system of equations (1871), Manning Formula (1889), the Thiessen polygon method to interpolate area average precipitation (1911), isochrones unit hydrograph method (1922), Pearson-III type curve method to fit the frequency distribution curve (1924), unit hydrograph method (1932), Muskingum method (1935), Synthesis Unit Hydrograph Method (1938), the Los Angeles hydrograph method (1944), the torrential rain intensity formula described using the exponential function (1950), the instant unit hydrograph of Clark method (1957), water ingress flood hydrograph method (1958), the Chicago method (1960), TRLL calculation program (1963), Muskingum-Cunge method (1969) and others. Those urban hydrological and hydraulic mathematical models are the foundation for simulations of trip difficulty for urban residents under rainstorm waterlogging conditions. 

Forecasts for urban rainstorm waterlogging early-warning systems play a key role in quick response to a disaster. Estimation of the trip difficulty for residents in the waterlogged area is a critical component for any disaster management plan. Japan is a country experiencing very frequent natural disasters. The country had to build a strong capacity to cope with urban waterlogging disasters. Presently in Japan, most of the urban rainstorm waterlogging studies use hydraulic tests to determine the safety thresholds for residents to walk across waterlogged areas, which is used to select the evacuation routes [4].

In a study by Daichi’s group, the authors developed a method to analyze the waterlogging data and to estimate the possibility for the residents to evacuate on foot during a disaster [5]. A comprehensive strategy was developed by also taking consideration human factors during the evacuation process. They also studied an evacuation process when basements were flooded by applying the hydraulic principles [6]; the pressure force exerted from water logging on doors per unit area was estimated, which, in combination with the flow rate and depth of the rainwater, was used to assess the trip difficulty for the residents to evacuate on foot. Then using a large scale in-door hydraulic test [7], the threshold values were determined for residents to walk across the waterlogged area, the length of time required to escape a car, and the relationship between water depth and the difficulty level for humans to escape a waterlogging disaster. In a study by Suga *et al*. [8,9] five factors, including the flood velocity, water depth, distance from disaster shelter, walking speed, and the awareness of risk in residents were compared, and the threshold values for residents to walk across waterlogged areas were estimated. 

In summary, several studies have been performed to assess residents’ trip difficulty level to evacuate on foot during an urban rainstorm waterlogging disaster. In most of the reported case studies, the disaster-affected objects and the surrounding environments were selected as the influential factors during the assessment procedure, whereas the dynamics of waterlogging process and simulation under different scenarios was not been fully investigated. 

In the present study, the process of development of an urban rainstorm waterlogging case was used initially to construct mathematical models which were then used to simulate the dynamics of urban rainstorm waterlogging formation. Factors such as water depth, flow directions, and water flow velocity were all embedded in the models, furthermore, irregular grids were used as skeletons for modeling which made the simulation more accurate. Additionally, scenario simulation methods were used to plot road waterlogging situations under different schemes of rainstorms, then locations of roads under waterlogging condition, the expanse of waterlogged areas, water depth, water flow velocity and directions were simulated dynamically. All of those factors were considered when assessing the trip difficulty for residents in waterlogged areas. The simulation results are very instructive for protecting the safety of residents and their propery, for directing the traffic, and for enhancing forecasting and early warning systems, thus taking appropriate actions to mitigate the risk of urban rainstorm waterlogging disasters. 

## 2. General Description of the Study Area

**Figure 1:**
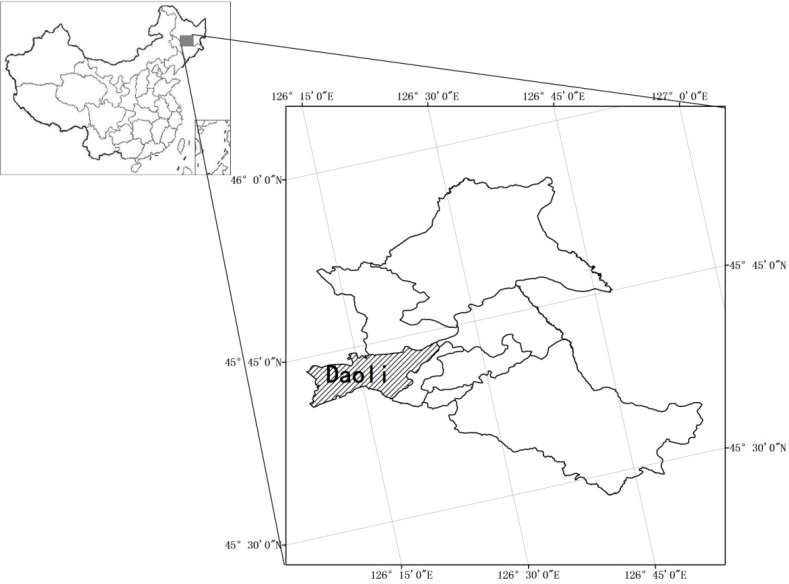
Diagram of the study area.

Ha-Erbin is the capital city of HeiLongjiang province in China, it is located at 125°42′–130°10′ east longitude and 44°04′–46°40′ north latitude (Figure 1). The city is the center of politics, economy, culture and transportation in Northeastern China, it has the largest metropolitan area in the region and is also the second largest provincial capital city in terms of expanse and population and one of the top 10 largest cities in China. The city has an area over 53.1 thousand square km, consisting of eight districts and 10 counties (suburbs). The total population is 10.635 million people, and downtown residents are over 5.879 million people. The average annual rainfall precipitation is 569.1 mm, summer is hot and humid with frequent rains. Major precipitation occurs in June–August, which receive over 60–70% of total annual rainfall. Because of the rainfall seasonality, rainstorms occur periodically during the summer.

In recent years, the city of Ha-Erbin has been growing rapidly. Neither the new nor the old towns have efficient drainage pipelines, and the cross sectional area of the drainage system is too small. According to the records, in 2011 the water discharge pipeline in the city was 993 km long. Drainage pipes only covered 66% of the area, leaving the remaining 34% of the urban area with no drainage outlets. What made it worse was that over 30% of the pipelines were outdated, and there were 27 km of pipelines that have been in use for over 70 years and the pipes were seriously aged. According to current national regulations, urban water discharge pipelines should have a discharge capacity of 185 m^3^/s to handle a medium-rainfall (25 mm/h precipitation), and the standard for urban rain water sewer line density is 11 km per square km, for rain water and sewer convergence discharge pipeline density it is 8 km per square km. In Ha-Erbin, the water discharge capacity was only 117 m^3^/s, which is 68 m^3^ lower than the medium-rain standard. As a consequence, when there was a medium-rainfall, rain water would accumulate on road surface at a rate of 68 m^3^/s, and within about half an hour, some roads started to have paddling-depth water.

In the city, rain water and sewage are discharged through the same pipelines. The discharge pipeline density was 5.36 km/square km, which is 30% below the national standard. In the old town, as population continued to increase, areas of water convergence also expanded. However, improvement of the water discharge pipelines fell far behind, and as a consequence some road sections would have water logging due to a lack of drainage system. As more household garbage was produced and continuously disposed of into the drainage pipe lines, some of the pipes became clogged, and the roadways were heavily flooded when it rained, affecting residents’ safety. 

## 3. Data Treatments and Methodology

### 3.1. Data Processing

Data used in this study were from the 1961 to 2007 rainfall precipitation records in Har-Erbin, data from high resolution remote images (at 0.61 m resolution), and information about the water discharge pipelines in the city. The city underlying surface basic information was extracted from remote image datasets, including road information, residential location information, ground surface roughness coefficients, area and other parameters. Data were processed using the spatial analysis tools provided in the ARCGIS software. 

#### 3.1.1. The Generalization of the Topology and Terrains

Construction of urban rainstorm waterlogging models requires large amounts of data on topology, terrains, water discharge systems and basic information about the city. The distribution of those waterlogging-causative factors has a significant impact on the level of the disaster, and urban topology and water discharge pipelines are complicated systems. Even after grid classification treatments the size of the information data was still quite large, which is not easy to handle in performing an accurate simulation. Therefore, all the data were subjected to generalization before being used for building models. The generalization principles included using grid as the basic calculation unit, and the position of a grid was determined by the horizontal coordinates of the centroid.

Grids and their edges were numbered, and the elevation of a grid was the mean of terrains in the respective grid. The corresponding underlying surface properties for each grid was used to assign the type and roughness coefficient of the grid; the ratio of the impervious area was used to correct the grid area. These data were used to construct the basic property dataset for a grid, and the information was stored in the property table. 

#### 3.1.2. Generalization from Basic Information on Urbanization Process

During urban construction, large parcels of lands previously covered with natural vegetation are now being replaced by impervious concrete roads. As a consequence, the water withholding and infiltration capacity at the ground level drops to a very low level, thus inducing serious runoff problems. The residential house foundations and road surface are normally raised above ground level, which increases slope discharge thus shortening the time period for water to converge at the ground level and meanwhile increasing the precipitation runoff coefficient in the urban area. 

It is obvious that urbanization has brought about alterations in the impervious surface areas as well as the slope gradient of lands. Those two factors are the main causes for changes in urban hydrologic properties, they are also responsible for the worsening urban rainstorm waterlogging situation in recent years. Therefore, the generalization of urbanization adopted the following principles: the ratio of impervious area in the grid (Axy) was used to represent the degree of urbanization; the definition of precipitation and convergence coefficient is: f = 0.4 + 0.44Axy; the amount of ground level runoff is: q = A × R × f, in which A is the grid area and R is the amount of rainfall per unit area of the grid.

#### 3.1.3. Generalization of the Water Discharge Systems

In downtown Ha-Erbin, the drainage system has three components: the discharge pipelines, pump stations, and submerged discharge flow pipes. Two types of drainage systems were in place. One system used the submerged discharge pipes to discharge water directly into rivers, and in the second one, water on the ground and in pipes was pumped through sewer lift stations before being discharged into the primary and secondary rivers. Therefore, for the generalization of water drainage systems, grid units were divided into two types: with or without pipelines. The diameter of the grid was the average value of diameters of all the pipes, and the length of the pipeline is the total length of all pipes. Grids with pump stations and submerged discharge pipes were designated as discharge outlets, and the discharge capacity for those grids were determined using the generalized equations.

### 3.2. Methodology

The downtown district in Ha-Erbin where heavy urban rainstorm waterlogging occurs frequently was the modeling object. The simulated urban rainstorm waterlogging models were first constructed and then data collected from an experimental test were applied to the model to determine the threshold values of trip difficulty for residents walking across the waterlogged roads. GIS was applied to model the waterlogging process after rainstorms under different scenarios, and to determine the residents’ trip difficulty level in downtown area. A visualization method was also developed. 

**Figure 2:**
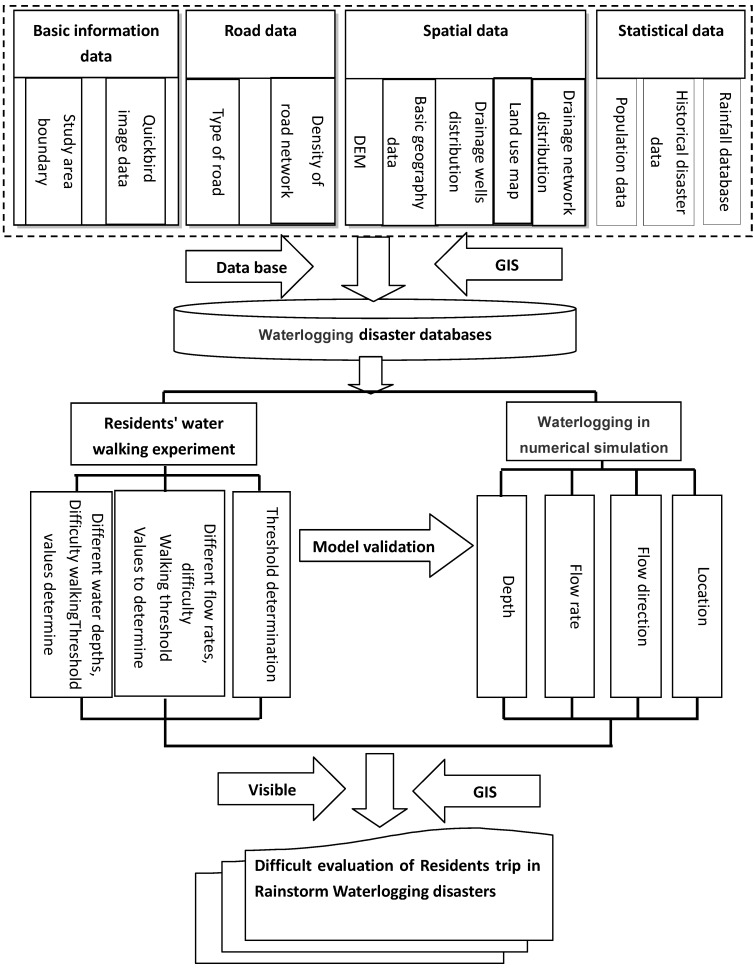
Technical flowchart.

Records of flood and precipitation data, and terrains and topographical properties of downtown area were used to reconstruct the waterlogging scenarios. The GIS technology was applied to improve the existing waterlogging models by simulating the depth and area of waterlogging and the velocity of water flow. The trip difficulty for urban residents under three reoccurrence scenarios of waterlogging conditions were determined using the GIS spatial analysis tools (Figure 2). 

#### 3.2.1. Construction of the Mathematical Models of Urban Rainstorm Waterlogging

**Figure 3:**
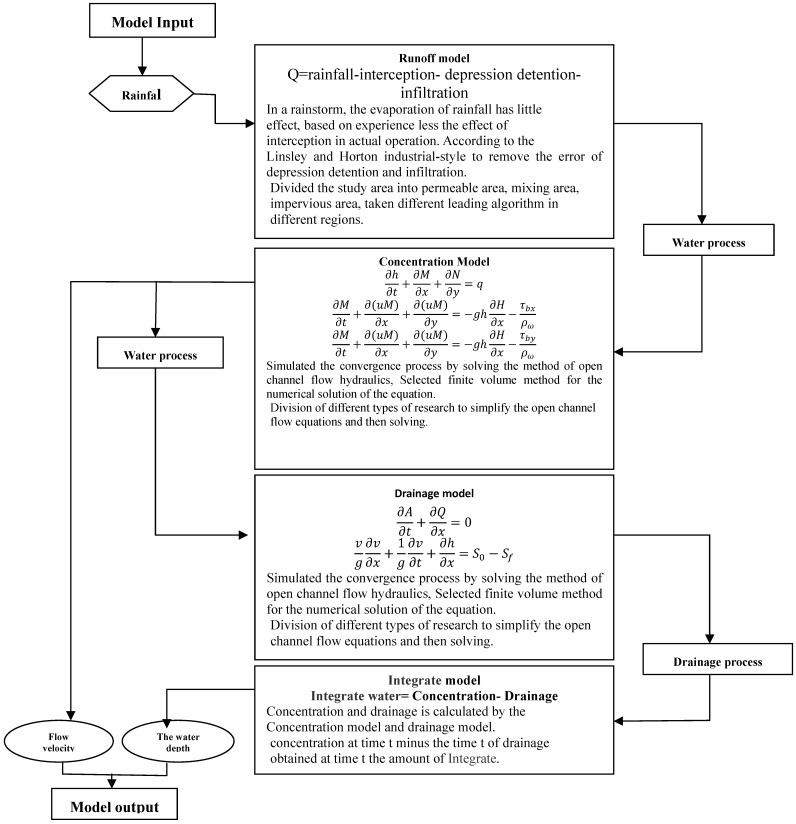
Flowchart of the numerical simulation models for treacherous factors in urban rainstorm waterlogging.

Several simulation methods can be used to model waterlogging situations on urban streets and roads, but most of them only use the depth of waterlogging as the determinant factor. None of the models include the velocity and direction of flowing water [10,11,12,13]. In this study, the impact from both the depth and flow velocity of waterlogging was considered. Models were constructed using the two-dimensional nonsteady flow as the skeleton, and irregular grids were derived from the source terrain data [14]. In the comprehensive urban rainstorm waterlogging mathematical model, water drainage pipelines were simulated using a one-dimensional nonsteady flow method [15,16,17,18]. The modeling system was comprised of four modules: runoff producing model, water convergence model, water drainage (discharge) model, and waterlogging model (Figure 3). The runoff producing model was processed using the hydrologic model method, the water convergence model was simulated using the hydrodynamics of runoff streams model; and the water discharge was simulated to fit generalized data; the waterlogging model was a comprehensive one combing the three aforementioned models. Among the four modules, the convergence model is the main body of the modeling system; it was constructed using the two-dimensional unsteady flow equation as the basis. In this model, based on land topology and features of terrains, the whole study area was divided into irregular grid cells. Computation of the depth and area of waterlogging was performed per grid unit using a finite volume method.

During the computational process, topography and terrain features were generalized into grid coordinates, such as the types of grids, elevation, roughness coefficients, area coefficient of correction, and water discharge capacity. The between-grids water exchange was calculated on the passage around the grids. For the secondary rivers at smaller spatial scales, they were generalized to special passages, and movement of waterlogging streams within these channels was calculated using the one-dimensional unsteady flow equation. The amount of water exchanges between those special channels and grids were calculated using the broad crested weir flow formulas.

The two-dimensional unsteady flow equations are: 

The continuous equations:


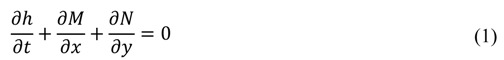


The momentum equation:


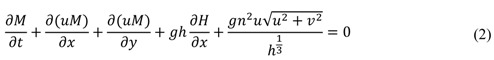


and:


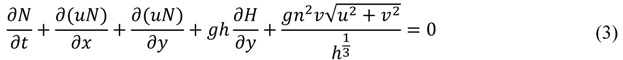


where *h* is the depth of water, *H* is the water level, *q* is the source-sink term representing the effective precipitation intensity in the model, *M* and *N* are the amounts of discharge per unit width in the *x* and *y* directions, *u and v* are the fraction of velocity in the *x* and *y* directions, *n* is the roughness coefficient, and *g* is the gravitational acceleration, and *t* is the time of water to grid channel.

The one-dimensional unsteady flow basic control equation is


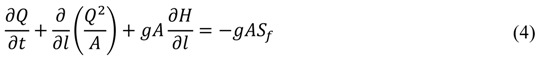


where Q is the amount of area flow, A is the area of cross section of flow, is gradient drop of friction, *t* is the time of water to grid channel, and *l* is the length of grid channel. 

The broad crested weir flow calculation equation is:


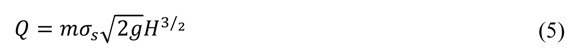


in which Q_j_ is discharge flow per unit dam top width, *m* is coefficient of overflow of broad crested weir, Q_s_ is the coefficient of submergence, and H_j_ is the water level of weir crest.

The flow rate calculation equation is:


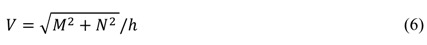


where *V* is the water flow velocity, *M* or *N* each is the amount of water flow per unit dam width in the *x* or *y* directions, and h is the depth of the accumulated water. 

#### 3.2.2. Extraction of the Directions of Waterlogging on Urban Streets

**Figure 4:**
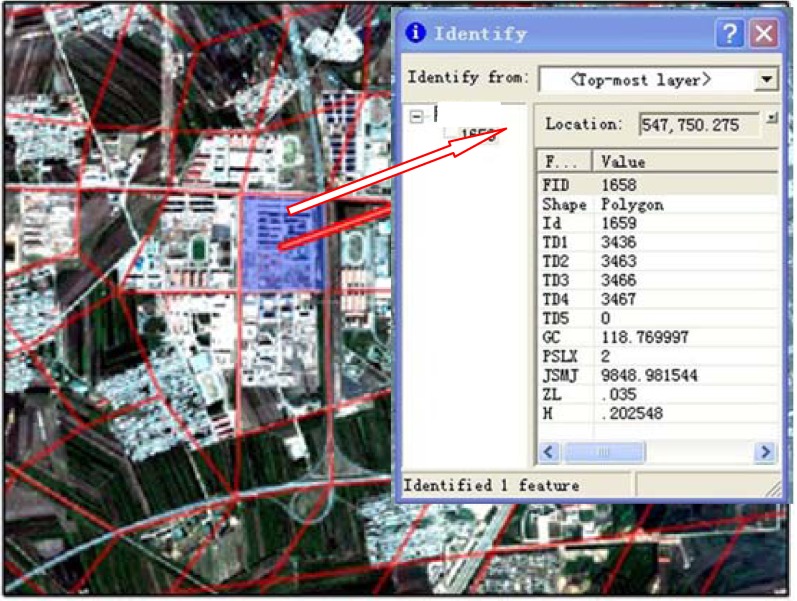
Grids, routes and properties in the models.

In this study, the DEM and hydrology toolset were used to extract data for the flowing direction and the volume of rainstorm water as well as the length of roads under waterlogging condition, and the river networks including grading of the system and factions of the drainage basin in the study area. These data were used to construct the surface water runoff model. After basic hydrology analysis of these data, an active process of flowing water was reconstructed on a digital elevation model (DEM) surface. Eventually a complete hydrological analysis process was simulated which allows for extraction of water flowing directions within grids (Figure 4). 

#### 3.2.3. Generation of Irregular Grid Cells

Generation of the terrain model is the most complex step in simulating urban rainstorm waterlogging. Special considerations have to be given to buildings and constructions which create continuous blockages to water flow, such as houses, dikes, above-ground level streets, highway bridges and train rails. In this study, no nodes were plotted for those structures. For the secondary rivers within urban limit lines, instead of assigning them into the special passage category, they were generalized into smaller grid cells that were scaled proportionally to the widths of the river. Properties of grids and passages were described for these features, as well as infrastructures blocking water flow and discharge facilities (Figure 4). 

According to the topography and features of the terrains, the area under simulation was divided into irregular grid cells which were the basic units for calculating the size of flooded area and water depth using the finite volume method [19,20,21]. Therefore, classification of grids is very critical for accurately simulating the urban rainstorm waterlogging. The basemap consists of the ArcMap layer, the contour line maps layer, drainage map, and the small streams layer derived from hydrologic analysis. Then the grids were classified aided with the QuickBird Image data, and the accuracy of grid classification was examined using TOPOLOGY ANALYSIS. 

Next, the data introperation tool in the ARCGIS data management wizard was activated to generate passage around the grids. The spatial association tool was used to construct the topological relationship between grids and passages, *i.e.*, using the grid ID to generate the passage ID around the grid, or *vice versa*, and properties of grids and passages were finalized. Grids and passages are all data object files, therefore they can store data, and those datafiles were assigned topological connections. Then using data from the contour line map, road network, river water system, and land constructions, the whole study area was generalized into 1,724 different sized irregular grids with an average area of 0.326 km^2^, and 3,572 passages of average of 0.587 km in length. 

#### 3.2.4. Establishing the Topology Relationship for Grids and Passages

The finite volume method was used to discretize the two-dimensional unsteady flow equation, and the results were used to classify grids and to establish the interaction among all the passages. Certain coding rules were applied to connect grids and passages, to clearly identify the passages for each grid, and the grids on both sides of a passage. In this study, the ARCGIS spatial analysis functions such as Conversion (Pint to Polygon/polyline) and grid intersection were used to set the topology for grids and passages. Results were converted into notepad format using the map attribute table before being exported to computation programs. 

### 3.3. Experimental Test of Trip Difficulty Thresholds for Acrossing Waterlogged Area

Trip difficulty refers to the situation encountered by residents walking across, or driving through, waterlogged areas after a short but heavy rainstorm in the urban area. In general, when water on roads reaches 0.3 m in depth, residents will walk at reduced speed; walking becomes very difficult in 0.5 m deep water, and it is impossible to walk across 1 m deep water. For vehicles driving in 0.3 m deep water, the engine will stop running when water get into the exhaust systems. 

#### 3.3.1. Experimental Methodology

In this study, the main objectives were to determine resident trip difficulty threshold values in logging water. The speed of walking in water was tested on elementary school children and young residents. Both groups have the average body sizes for the respective age populations at the national level (Table 1). The experimental methods were set up by letting participants walk in upstream and downstream directions of the current while water depth and flow velocity were recorded. The trip difficulty threshold levels were determined accordingly. 

**Table 1:** Information of the participants.

Identity	Age	Weight (Kg)	Height (cm)	No of residents	Walking distance (m)	Toad type	Walking direction
Pupil	8–12	30–45	125–155	10	15	Asphalt	Downstream, upstream
Youth	18–24	55–75	168–175	10	15	Asphalt	Downstream, upstream

#### 3.3.2. Experimental Results

For the safety of the participants, extreme waterlogging situations were not simulated in this study. The experimental tests show (Table 2) that walking slowed down as it got more difficult in deeper and faster flowing water. 

**Table 2:** Information of the experimental participants.

		Pupil					Youth		
Number	Velocity m/s	Depth m	Distance m	Speed m/s	Number	Velocity m/s	Depth m	Distance m	Speed m/s
A1	0.00	0.35	4.50	0.91	C1	0.48	0.30	15	0.84
A2	0.35	0.35	4.50	0.73	C2	0.78	0.20	15	0.90
A3	0.48	0.35	3.00	0.85	C3	0.78	0.30	15	0.88
A4	0.25	0.42	4.50	0.66	C4	0.50	0.50	15	0.58
A5	0.50	0.20	4.50	0.56	C5	0.50	0.70	15	0.56
A6	0.50	0.20	4.50	1.00	C6	1.00	0.40	15	0.68
A7	0.77	0.20	4.50	0.98	C7	1.30	0.30	15	0.78
A8	0.48	0.20	4.50	0.87	C8	1.20	0.60	15	0.48
A9	0.48	0.57	4.50	0.56	C9	0.95	1.00	15	0.34
A10	0.48	0.60	0.30	0.58	C10	0.95	0.50	15	0.53
B1	0.48	0.35	7.50	1.02	D1	1.10	0.20	15	0.90
B2	0.77	0.35	7.50	0.90	D2	1.20	0.30	15	0.81
B3	0.48	0.35	7.50	0.84	D3	1.30	0.40	15	0.88
B4	0.48	0.40	7.50	0.70	D4	1.10	0.30	15	0.85
B5	0.77	0.35	7.50	1.20	D5	1.20	0.40	15	0.88
B6	0.48	0.30	7.50	0.85	D6	1.30	0.50	15	0.78
B7	0.36	0.35	7.50	1.23	D7	0.95	0.70	15	0.30
B8	0.36	0.25	7.50	1.10	D8	0.60	0.40	15	1.00
B9	0.77	0.25	7.50	0.88	D9	1.41	0.80	15	0.53
B10	0.48	0.50	7.50	0.76	D10	0.57	0.60	15	0.68

Note: A1–A10 is K-12 students walking against current, B1–B10 are students walking with the current, C1–C10 are youth walking against the current, D1–D10 are youth walking with the current.

**Figure 5:**
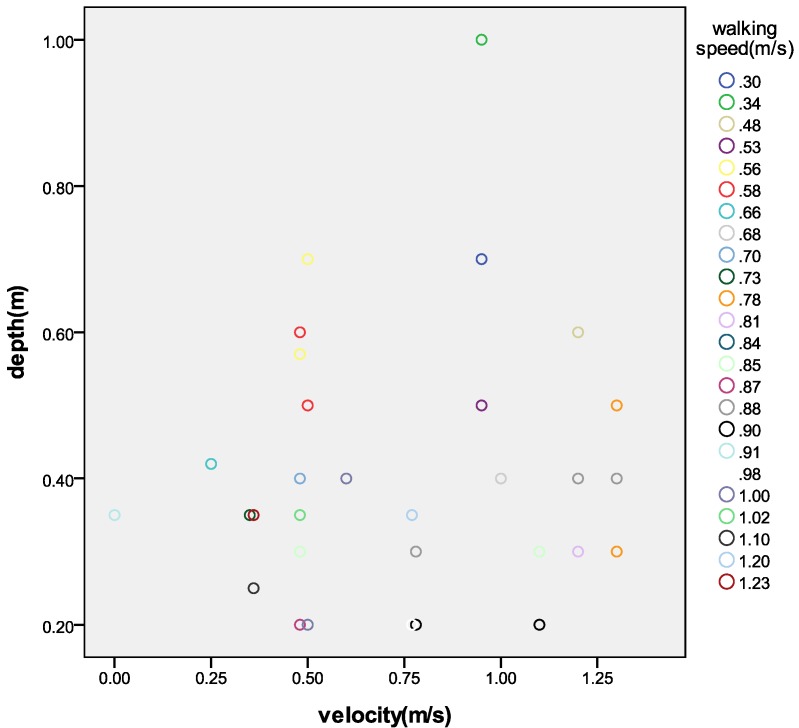
Relationship between walking speed and the depth and velocity of water flow.

These results confirmed that that the flow velocity and depth of water both have a profound effect on urban residents travelling under rainstorm waterlogging conditions. Water of 0.1 m–0.3 m in depth and flowing at 0.1 m/s–0.5 m/s had no obvious effect on walking (Figure 5). When water reached 0.5 m–1.0 m and was flowing at 0.5 m/s–1.0 m/s, walking in the water became very difficult. When water reached 1.0 m deep and was flowing over 1.5 m/s, walking was severely impeded. Around deep waterlogged areas residents would not be able to travel as roads became impassible.

**Figure 6:**
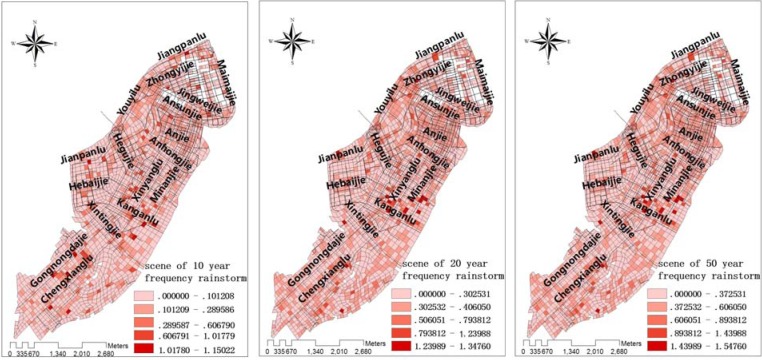
Depth of accumulated water in urban area under three scenarios: from right to left: occurring every 10 years, 20 years, 50 years.

## 4. Results and Discussion

### 4.1. Simulation and Visualization of Urban Waterlogging Scenarios

**Figure 7:**
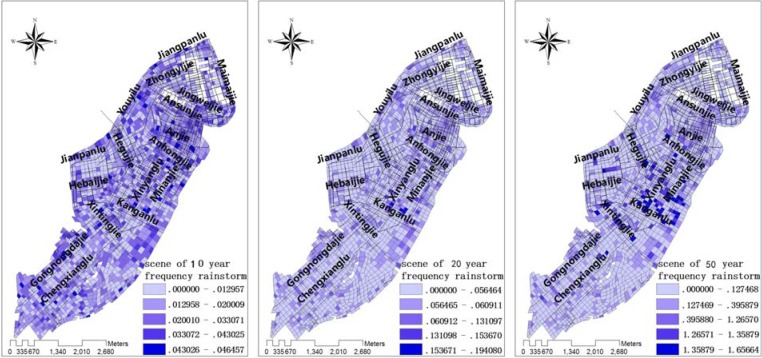
The velocity of waterlogging under three scenarios of reoccurrence of rainstorm: from right to left: every 10 years, 20 years, 50 years.

**Figure 8:**
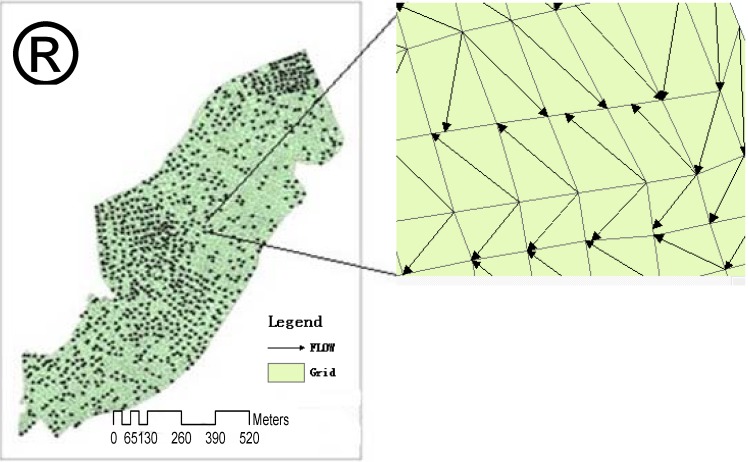
The flowing direction of waterlogging after rainstorm in urban area.

Urban waterlogging scenarios were simulated for road conditions under various rainstorm conditions. After fitting the historical data of urban rainstorm waterlogging, models were constructed for three scenarios which would occur once every 10, 20 or 50 years. Then data for the waterlogged locations, and the depth and flowing velocity and directions of water, were generated to construct the GIS topic maps for the city (Figure 6, Figure 7, Figure 8).

### 4.2. The Relationship between Trip Difficulty and the Depth and Flow Velocity of Waterlogging

Options of accessible-roads were used as major criteria for assessing the trip difficulty in urban rainstorm waterlogging, because waterlogged roads would be impassable to residents, thus threatening the mobility and safety of residents. The major factors affecting road accessibility are the location of the waterlogged streets, the size of the area under water, and the depth, flow velocity and direction of water. After combining information from historical record data and results from the experimental tests conducted in this study as well as referring to published papers [23,24,25], it was concluded that when water on the road is 0.5 m deep and flowing at 1.0 m/s, walking across water becomes very difficult. When water reaches above 1 m deep and moving at 1.5 m/s, the roads would be inaccessible to residents [26,27]. The difficulty levels of walking across areas with different depths and flowing rate of waterlogging were assessed (Table 3). 

**Table 3:** Relationship between trip difficulty for residents and the depth of waterlogging.

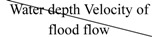	*h* < 0.5 m	0.5 m < *h* < 1 m	*h* > 1 m
*v* < 0.5 m/s	Passable	Passable	Difficult
0.5 m/s < *v* < 1.5 m/s	Passable	Difficult	Impassable
*v* > 1.5 m/s	Difficult	Impassable	Impassable

### 4.3. Assessment of the Resident Trip Difficulty and the Visualization Results

Using the simulated results under the three unban rainstorm waterlogging scenarios described above, subject maps were generated for the waterlogging depth and flow velocity using ARCGIS. Then the interpolation and buffer wizards in the conversion tools were activated to import the simulated data for water depth and flow rate to obtain the trip difficulty threshold value for each of the grids.

Based on the experimental results, the historical records and the situation in downtown Ha-Erbin, the trip difficulty in urban rainstorm waterlogging conditions was divided into three levels: passable roads (water depth 0 m-0.5 m, and flow velocity at 0 m/s-0.5 m/s), difficult but passable roads (water depth at 0.5 m-1 m and flow velocity at 0.5 m/s-1.5 m/s), and impassable roads (water deeper than 1m and flowing velocity above 1.5 m/s). 

**Figure 9:**
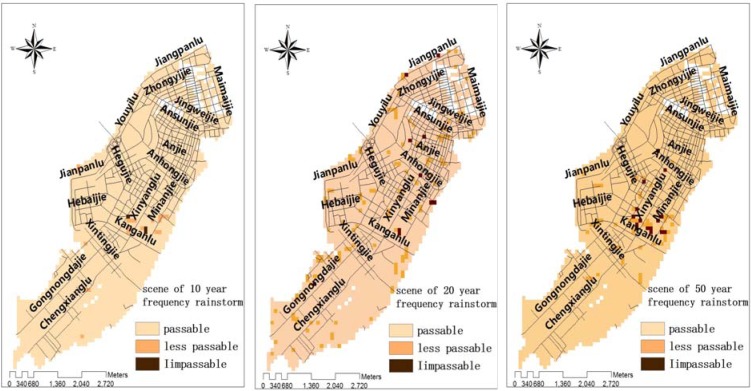
Evaluation of resident trip difficulty under the three scenarios.

From Figure 9, it can be seen that in the urban rainstorm waterlogging scenario which reoccurs once every 10 years, only three grids were impassable for residents, while other areas remained open. Under this scenario, the safety of residents travelling on foot and traffic flow should be normal. In the once-every-20-years-scenario, 13 grids were impassable, while other areas were open to the traffic. This situation would have some impact on the mobility of residents, traffic jams could occur on some waterlogged roads. In the once-every-50-years scenario, there were 86 grids that would become impassable to residents, while the rest of the city were still open. This situation would have caused traffic jams in expanded areas, roads could be closed for residents and vehicles. Emergency response measures should be taken by the respective agencies in charge to resolve the traffic problems. 

### 4.4. Discussion

This study was performed in the downtown (Daoli) district in Ha-Erbin. The two-dimensional unsteady flow was used as the basic controlling equation, and irregular grids as the basic skeletons. A “three-layer model” was constructed to simulate various urban rainstorm waterlogging scenarios. The model was put into a test on 10 August 2011 in a strong thunderstorm rainfall caused by the north-bound hurricane “Muifa” in Ha-Erbin, and it was proven effective. Meanwhile, according to the rainstorm characteristics in Ha-Erbin, the model was used to simulate the resident trip difficulty in urban rainstorm waterlogging scenarios that reoccur once every 10 years, 20 years, or 50 years. 

Urban rainstorm waterlogging can be very treacherous in certain areas. Together with the fast moving speed and rapid-spreading properties of precipitation from rainstorm, it is hard to develop an efficient strategy to control the waterlogging situation. Deploying an inappropriate approach can actually exacerbate the resultant damage. After waterlogging spreads into large areas, it will become very difficult to evacuate residents or divert traffic, it also makes it harder for the rescue workers to reach the disaster-stricken areas. 

In this study, several small-scale urban rainstorm waterlogging scenarios were assessed for the consequential effects on trip difficulty for residents. The research can be expanded to the assessment of urban basic construction, transportation roads and systems, and hazardous disasters in the ecological systems. The simulation methods are applicable in the prediction, forecast and validation of different types of natural disasters on regional scales, the construction of the vulnerable curves in response to natural disasters in urban area, and the enhancement of rapid response to emergency waterlogging situation in urban areas. Application of the technology will benefit rapid assessment of potential disaster damages, improving the level of management and mitigation of risks against natural disasters. 
